# Evaluation of Head Injury Criteria for Injury Prediction Effectiveness: Computational Reconstruction of Real-World Vulnerable Road User Impact Accidents

**DOI:** 10.3389/fbioe.2021.677982

**Published:** 2021-06-29

**Authors:** Fang Wang, Zhen Wang, Lin Hu, Hongzhen Xu, Chao Yu, Fan Li

**Affiliations:** ^1^School of Automotive and Mechanical Engineering, Changsha University of Science and Technology, Changsha, China; ^2^School of Mechanical and Automotive Engineering, Xiamen University of Technology, Xiamen, China; ^3^State Key Laboratory of Advanced Design and Manufacturing for Vehicle Body, Hunan University, Changsha, China

**Keywords:** head injury criterion, injury prediction, vulnerable road user, impact accident reconstruction, computational biomechanics model

## Abstract

This study evaluates the effectiveness of various widely used head injury criteria (HICs) in predicting vulnerable road user (VRU) head injuries due to road traffic accidents. Thirty-one real-world car-to-VRU impact accident cases with detailed head injury records were collected and replicated through the computational biomechanics method; head injuries observed in the analyzed accidents were reconstructed by using a finite element (FE)-multibody (MB) coupled pedestrian model [including the Total Human Model for Safety (THUMS) head–neck FE model and the remaining body segments of TNO MB pedestrian model], which was developed and validated in our previous study. Various typical HICs were used to predict head injuries in all accident cases. Pearson’s correlation coefficient analysis method was adopted to investigate the correlation between head kinematics-based injury criteria and the actual head injury of VRU; the effectiveness of brain deformation-based injury criteria in predicting typical brain injuries [such as diffuse axonal injury diffuse axonal injury (DAI) and contusion] was assessed by using head injury risk curves reported in the literature. Results showed that for head kinematics-based injury criteria, the most widely used HICs and head impact power (HIP) can accurately and effectively predict head injury, whereas for brain deformation-based injury criteria, the maximum principal strain (MPS) behaves better than cumulative strain damage measure (CSDM_0.15_ and CSDM_0.25_) in predicting the possibility of DAI. In comparison with the dilatation damage measure (DDM), MPS seems to better predict the risk of brain contusion.

## Introduction

Traumatic brain injury (TBI) has become a global health problem due to its corresponding high fatality and disability rates (Corrigan et al., 2010). Statistics show that about 10 million people suffer from TBI each year worldwide (Fahlstedt et al., 2016). Deaths due to TBI were reported to account for 40% of all deaths annually, and TBI is the main reason for mortality under the age of 45 in the United States. The incidence of TBI in the population of young people (15–30 years) was 154–415/100,000 in the United States, 535/100,000 in France, and 240/100,000 in Australia (Popescu et al., 2015). Currently, there is no ongoing large-scale epidemiological investigation of TBIs in China; however, according to statistics based on the national TBI database, the mortality rate of patients hospitalized for TBI is known as 27.23% (Yang et al., 2017). TBIs not only bring immeasurable pain to patients but also cause huge losses to the whole society. The main causes of TBI are traffic accidents, falls, and attacks, with traffic accidents being the second largest cause of TBI (Gabler et al., 2016; Hu et al., 2020, 2021). Therefore, studies on TBIs in traffic accidents have great practical significance.

In order to reduce the risk of brain injury from traffic accidents and other impact loads, a great deal of knowledge on the biomechanics of brain injury has been accumulated through research work, in which different injury evaluation criteria for different types of head injuries were also proposed. These criteria were initially derived from the well-known Wayne State Tolerance Curve (WSTC; Lissner et al., 1960) and is based on human cadaver head impact tests, which show the relationship between the average acceleration of head movement and its duration (Antona-Makoshi, 2016). Based on the WSTC, the severity index (SI) was later proposed by Gadd (1966), and Versace further modified the SI as a head injury criterion (HIC; Versace, 1971a). In 1974, the US government included HIC in Federal Motor Vehicle Safety Standard 208, which is the only HIC so far that is widely used in global automotive safety regulations. However, as the HIC only considers linear acceleration and action time and does not regard the rotational movement of the head, its deficiencies are gradually pointed out (Gabler et al., 2016). Over a decade later, Newman et al. proposed the Generalized Acceleration Model for Brain Injury Threshold (GAMBIT; Newman, 1986). Moreover, the head impact power (HIP) was proposed on the basis of GAMBIT a few years later (Newman et al., 2000). Both of these criteria consider both the linear and rotational accelerations of the head. With the expansion of in-depth research on the mechanism of head injury, researchers have put forward many head injury evaluation criteria, among which two representative ones are the rotational injury criterion (RIC) proposed by Kimpara and Iwamoto (2012) and the brain rotational injury criterion (BrIC) presented by Takhounts et al. (2013).

At present, the evaluation criteria of head injury are mainly divided into two categories: one is based on head kinematics response, and the other relies on brain tissue deformation response; all of the above-mentioned HICs utilize the head kinematics response. Head injuries have essentially two types, skull fracture and brain injury, with the latter divided into local brain injury and diffuse brain injury. Local brain injury includes contusion, acute subdural hematoma (SDH), epidural hematoma (EDH), and subarachnoid hemorrhage. The main manifestations of diffuse brain injury are concussion and diffuse axonal injury (DAI). The principal causes of the above injuries include concentrated pressure, intracranial viscous load, and craniocerebral inertial load (Yang, 2005), which can also be considered as collision force factors and inertia factors (including linear acceleration and rotational acceleration). In view of these common brain injuries, researchers have established corresponding injury criteria to effectively evaluate various injury types, such as cumulative strain damage measure (CSDM; Takhounts et al., 2003, 2008, 2013; Gabler et al., 2016) and maximum principle strain (MPS; Takhounts et al., 2008; Gabler et al., 2016) for the evaluation of DAI, or dilatation damage measure (DDM; Takhounts et al., 2003) and MPS (Bain and Meaney, 2000) to evaluate contusion. The relative motion damage measure (RMDM) is used to assess SDH (Takhounts et al., 2003; Gabler et al., 2016).

Biomechanical experiments have played a significant role in the development of these HICs (Nahum et al., 1977; Al-Bsharat et al., 1999). However, the subjects of biomechanical experiments are mostly animals and postmortem human subjects (PMHSs), causing deviations of the experimental measurement accuracy. Moreover, the loading conditions of human and animal cadavers and the consequent injuries are significantly different from those in traffic accidents (Kleiven, 2007; Gabler et al., 2016). Traffic accident reconstruction can provide researchers with more realistic injury data, thus making up for the lack of real information in this area. Therefore, many researchers believe that traffic accident reconstruction is one of the most effective methods to study head and brain injuries (Kleiven, 2007; Li and Yang, 2010). With the rapid development of computer technology and the computational biomechanics model of the human body, traffic accident reconstruction has become a common tool to study the complex biomechanical response of the head due to impact (Miller et al., 1998; Hu et al., 2007; Gabler et al., 2016; Wittek et al., 2016; Wang et al., 2018; Li et al., 2019). In previous studies on vulnerable road user (VRU) head injuries in traffic accidents, multi-rigid body [or multibody (MB)] models were mostly used for accident reconstruction and injury analysis (Lyons and Simms, 2012; Li et al., 2017; Shi et al., 2018); however, the MB models were only able to obtain head kinematics response and head kinematics-based injury parameters but not brain tissue injury parameters. Although a few studies (Katsuhara et al., 2014) have used finite element (FE) models to simulate collisions between vehicles and VRUs, such methods have high time cost and low adjustment flexibility, which in turn affects the efficiency of accident reconstruction. In order to shorten the calculation time, other researchers (Marjoux et al., 2008; Li and Yang, 2010) used an FE windshield and human head model to simulate the impact process between human head and windshield, and the boundary condition of collision is based on the result of a kinematics reconstruction with MB models. Obviously, although this method can obtain the brain tissue injury parameters and improve the calculation efficiency, it ignores the influence of other body segments on head injury (Ruan et al., 2007; Gabler et al., 2016; Jones et al., 2016; Wang et al., 2018).

In view of the above deficiencies, we proposed a coupled FE–MB human body model [coupled pedestrian computational biomechanics model (CPCBM)] in our previous study (Yu et al., 2020), where it was confirmed that the risk of brain injury in an accident is lower than the real injury when only the head model (i.e., head-only model) is used to reconstruct the accident (Wang et al., 2020). Therefore, the coupled FE–MB human body model is used in the present study to reconstruct the accident and reproduce the head injury. In addition, the applicability and effectiveness of the HIC is subsequently analyzed and evaluated to assess the head injury in traffic accidents with the aim to reduce the risk of head injury of VRUs in traffic accidents.

## Materials and Methods

### Research Protocol

**FIGURE 1:**
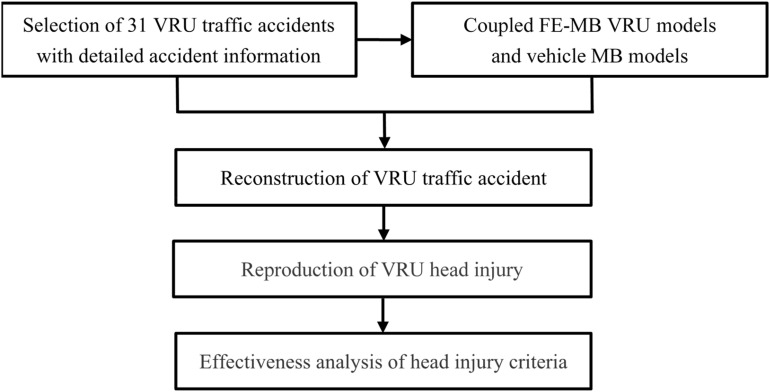
Schematic of the research process.

The schematic of the procedures performed in the present study is shown in Figure 1. First, 31 real-world VRU traffic accidents with detailed accident information were selected from the traffic accident database (section “Accident Data”), and the computational modeling of the accident participants is completed (section “Model Description”). Second, VRU traffic accident reconstruction and VRU injury replication (section “Accident Reconstruction”) were carried out. Finally, the effectiveness of HICs in predicting head injury in VRU traffic accidents was analyzed (sections “Analysis of Head Kinematics-Based Injury Criteria” and “Analysis of Injury Criteria Based on Brain Tissue Deformation”).

All FE computations in this study were conducted using the LS-DYNA R10.0 non-linear explicit dynamics code by Livermore Software Technology Corporation LSTC (Livermore, CA, United States)^1^. Explicit dynamics analysis is an extremely popular method for FE models of injury biomechanics (Yang et al., 2011). The MB models were implemented using the MADYMO V7.7 MB analysis package by TASS (Helmond, Netherlands)^2^, which is widely used in injury biomechanics. The interfacing between the FE and MB models was performed using the coupling assistant module/function of the MADYMO MB analysis package.

### Accident Data

The VRU traffic collision accidents analyzed in this study were selected from the In-Depth Investigation of Vehicle Accident in Changsha (IVAC) database (Kong and Yang, 2010). This database was established by Hunan University in 2006, which has conducted comprehensive, in-depth, and systematic accident investigation activities in Changsha, China, and carried out detailed research on traffic accidents and subsequent human injuries. It is a highly valued database widely used by researchers to study the biomechanics of human injury, the epidemiology of traffic injury, and road traffic safety (Kong and Yang, 2010; Li and Yang, 2010; Nie and Yang, 2014, 2015).

In the present study, 31 typical vehicle-to-VRU impact accidents were selected from the IVAC database, among which accident Cases 1–17 were pedestrian impact accidents and accident Cases 18–31 were two-wheeler impact accidents. The selection process was based on the following criteria:

1. VRU impacts with the vehicle;
2. VRU head impacts with the front windshield of the car; and
3. head injury occurs in the accident.

The VRU and vehicle information of the selected 31 accidents is shown in Table 1.

**TABLE 1:** Basic information of 31 road traffic accidents selected for this study.

Case ID	VRU information					Vehicle information			Impact velocity (km/h)	
			
	Type	Gender	Stature (cm)	Weight (kg)	Age	Brand and model	Weight (kg)	Size (mm)	Vehicle	VRU
1	Pedestrian	Male	171	80	17	Volkswagen Jetta	1,490	4,428 × 1,660 × 1,420	30.0	2.1
2	Pedestrian	Male	172	60	20	Honda Accord	1,442	4,814 × 1,821 × 1,463	17.1	1.1
3	Pedestrian	Male	174	70	50	Volkswagen Jetta	1,490	4,428 × 1,660 × 1,420	37	0
4	Pedestrian	Male	173	68	63	Volkswagen Golf	1,275	4,400 × 1,735 × 1,470	43.2	5.0
5	Pedestrian	Male	176	76	35	Mercedes E-Class	1,455	4,800 × 1,800 × 1,400	46.8	7.0
6	Pedestrian	Male	180	77	57	Opel Astra	1,150	3,817 × 1,646 × 1,440	37.4	1.0
7	Pedestrian	Male	153	61	89	Volkswagen Passat	1,850	4,669 × 1,740 × 1,466	58.7	3.2
8	Pedestrian	Male	170	55	42	Volkswagen Jetta	1,490	4,428 × 1,660 × 1,420	43.6	6.5
9	Pedestrian	Male	176	75	50	Volkswagen Tiguan	1,545	4,506 × 1,809 × 1,685	48	5
10	Pedestrian	Male	174	75	52	Volkswagen Passat	1,590	4,789 × 1,765 × 1,470	40	5
11	Pedestrian	Male	166	65	70	Volkswagen Lavida	1,285	4,608 × 1,743 × 1,465	36	0
12	Pedestrian	Male	159	50	72	Volkswagen Polo	1,270	4,187 × 1,650 × 1,465	35	0
13	Pedestrian	Male	168	75	68	BYD F3	1,170	4,325 × 1,705 × 1,490	30	3.6
14	Pedestrian	Female	154	48	78	Zotye T600	2,000	4,648 × 1,893 × 1,686	36	0
15	Pedestrian	Male	175	70	56	Volkswagen Passat	1,850	4,789 × 1,765 × 1,470	70	5
16	Pedestrian	Male	158	55	79	Chevrolet Aveo	1,210	4,399 × 1,735 × 1,517	55	3
17	Pedestrian	Male	170	60	79	Hyundai Elantra	1,348	4,543 × 1,777 × 1,490	91	12
18	Cyclist	Female	157	60	55	Mazda Axela	1,286	4,461 × 1,795 × 1,474	30	10.5
19	Cyclist	Male	168	67	63	Geely Meiri	1,270	4,150 × 1,620 × 1,450	30	15.8
20	Cyclist	Male	170	60	54	Dongfeng Sokon	1,576	3,795 × 1,560 × 1,925	16.5	9.7
21	Cyclist	Male	170	80	58	Volkswagen Santana	1,540	4,595 × 1,750 × 1,430	34.7	7.2
22	Cyclist	Male	175	70	57	Iveco	2,325	4,845 × 2,000 × 2,500	40	4.3
23	Cyclist	Male	170	65	67	BAIC Hyosow S3	1,335	4,380 × 1,730 × 1,760	40.3	18.7
24	Cyclist	Male	158	49	65	Volkswagen Santana	1,540	4,595 × 1,750 × 1,430	31	5.5
25	Cyclist	Female	158	48	42	Volkswagen Santana	1,540	4,595 × 1,750 × 1,430	22	7.2
26	Cyclist	Male	165	60	65	Audi A4L	1,565	4,818 × 1,843 × 1,432	35	4.3
27	Cyclist	Female	161	45	23	Volkswagen Santana	1,540	4,595 × 1,750 × 1,430	34.7	0
28	Cyclist	Male	165	55	62	Volkswagen Jetta	1,500	4,428 × 1,660 × 1,420	40	7.2
29	Cyclist	Female	152	55	50	Wu Ling Sunshine	1,030	3,730 × 1,510 × 1,860	70	10.6
30	Electric two-wheeler	Female	155	40	13	Mitsubishi Outlander	1,500	4,695 × 1,810 × 1,680	35	3.6
31	Electric two-wheeler	Male	173	75	43	Hyundai Elantra	1,236	4,542 × 1,775 × 1,490	60	10.8

*VRU, vulnerable road user.*

According to the accident information recorded in the IVAC database, head injury rating data were obtained for the 31 selected accident cases, as shown in Table 2.

**TABLE 2:** VRU head injury rating information for the road traffic accidents subject to this study.

Case ID	Head injury		
	
	DAI AIS	Contusion AIS	MAIS
1	2	–	2
2	1	–	1
3	3	4	4
4	0	–	0
5	2	–	2
6	3	2	3
7	4	–	4
8	–	3	4
9	–	2	2
10	–	–	4
11	–	3	3
12	–	5	5
13	–	–	5
14	–	–	1
15	4	–	4
16	–	–	1
17	–	–	5
18	–	–	6
19	–	–	0
20	–	–	0
21	–	–	1
22	–	2	2
23	–	–	6
24	–	–	1
25	–	–	2
26	–	–	6
27	–	–	1
28	–	–	1
29	–	–	6
30	–	–	5
31	–	–	5

*VRU, vulnerable road user; DAI, diffuse axonal injury.*

*AIS, Abbreviated Injury Scale, which is the most widely used injury scale to quantify the injury severity of the human body organs/segments (AAAM, 2008).*

*MAIS, Maximum Abbreviated Injury Scale; maximum AIS scores of all types of head injury.*

### Model Description

#### Coupled Finite Element–Multibody Human Body Model

The numerical model of the human body used for accident reconstruction in this study is composed of an MB model and an FE model, which consists of the MB model of the 50th percentile adult male pedestrian model developed by TNO (The Netherlands Organization for Applied Scientific Research^3^) (TASS, 2013a,b,c), and the FE head–neck complex of the Total Human Model for Safety (THUMS) (Version 4.01) of the 50th percentile adult male by Toyota Central R&D Laboratories^4^ (Shigeta et al., 2009; Watanabe et al., 2011).

**FIGURE 2:**
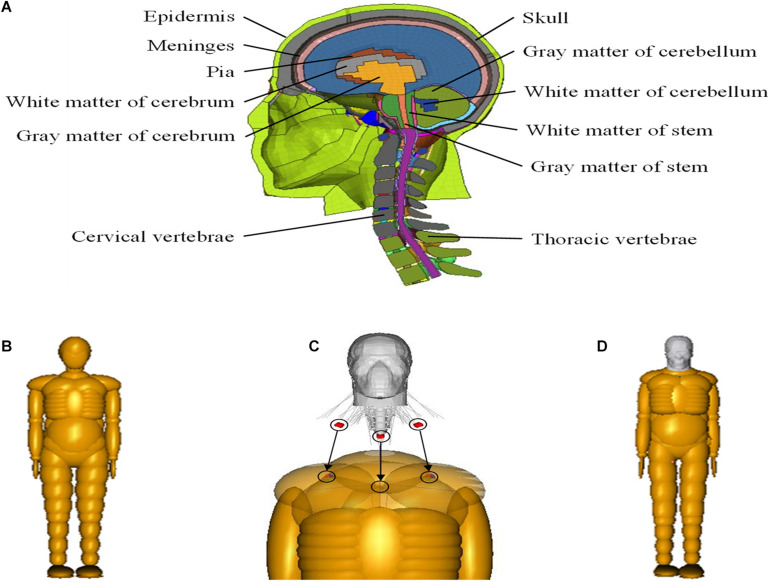
**(A)** THUMS head–neck FE model; **(B)** TNO 50th percentile adult male model; **(C)** coupling process between FE and MB models; and **(D)** coupled FE–MB human body model. FE, finite element; MB, multibody.

Due to the complex anatomical structure of the brain, biomechanical responses to head injury in VRUs involved in road traffic accidents cannot be simulated by the MB model, whereas the FE model with detailed structure is more useful in this regard. The current study uses the head and neck model of the widely used THUMS. The THUMS FE head model includes the key anatomical structures of the human brain, such as the scalp, skull, meninges, cerebrospinal fluid, brain, cerebellum, brain stem, falx, and tentorium, as shown in Figure 2A.

The TNO 50th percentile adult male pedestrian model is composed of 52 rigid bodies (Figure 2B), which are connected by kinematics hinges to simulate the stiffness characteristics of human tissues and joints. In order to simulate the interaction between various parts of the human body, as well as the interaction between the human body and the external environment, 64 ellipsoid surfaces and two planes attached to the rigid bodies are used to represent the outer body surface, and the contact characteristics are set for the rigid body surface of different parts.

For the coupling of the two models, the head and neck of the MB model are first removed, and the head–neck complex of the THUMS model and the remaining body segments of TNO pedestrian model are connected by using the coupling assistant module in MADYMO. The end node of muscle and cervical vertebra unit, originally connected with the trunk of THUMS model, are connected to the corresponding rigid bodies (left clavicle, upper torso, and right clavicle), as shown in Figure 2C. The coupled FE–MB human body model is shown in Figure 2D. For more detailed information about the coupled model, the reader is referred to our previous publications (Wang et al., 2020; Yu et al., 2020).

#### Bicycle Model

**FIGURE 3:**
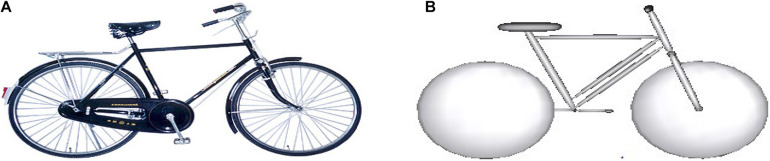
Modeling of the bicycle involved in Case 21: **(A)** the bicycle in the real-world accident; and **(B)** MB model of the bicycle. MB, multibody.

The bicycle model involved in the selected cases is established based on the information recorded in the accident investigation and the corresponding actual geometric information. Take Case 21 as an example: the developed bicycle model consists of five rigid bodies, including front wheel, rear wheel, frame, front fork, and pedal, connected to each other by hinges (Figure 3). The mechanical characteristics of each part are used following the literature (Nie et al., 2015).

#### Electric Two-Wheeled Vehicle Model

**FIGURE 4:**
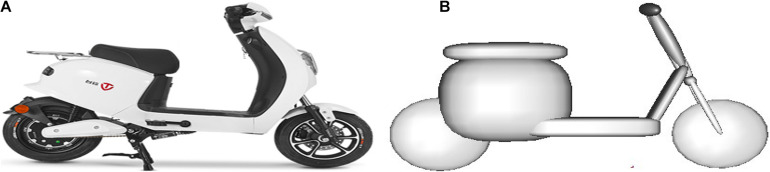
Modeling of the electric two-wheeler involved in Case 31: **(A)** the ETW in the real-world accident and **(B)** MB model of the ETW. ETW, electric two-wheeler; MB, multibody.

The modeling of the electric two-wheeler (ETW) is similar to the bicycle model. Take Case 31 as an example, where the ETW model consists of four rigid bodies, which are connected by hinges (Figure 4), including front wheels, rear wheels, frame, and front forks. The mechanical contact characteristics of each part have been studied and verified by predecessors (Deguchi, 2003).

#### Vehicle Model

**FIGURE 5:**
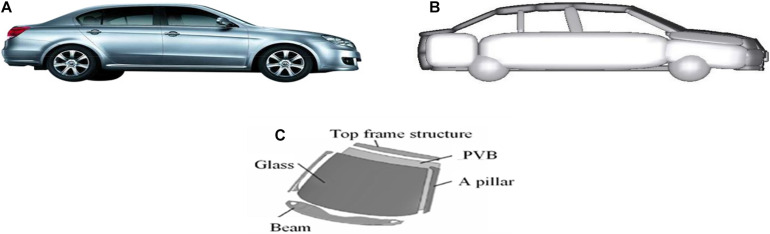
**(A)** The vehicle involved in the real-world accident; **(B)** MB model of the vehicle; and **(C)** FE front windshield model. MB, multibody; FE, finite element.

The vehicle model used in accident reconstruction consists of two parts: the MB vehicle model and the FE front windshield model. The MB vehicle model is established based on the structure and size of each part of the vehicle in a real accident, with the car involved in Case 11 for the example seen in Figures 5A,B. The FE front windshield model is composed of glass and the surrounding metal frame, and the glass model is composed of two shell elements: glass and the other polyvinyl butyral (PVB; Figure 5C); the corresponding material parameters and modeling methods have been verified in the literature (Yao et al., 2008; Li and Yang, 2010).

### Accident Reconstruction

**FIGURE 6:**
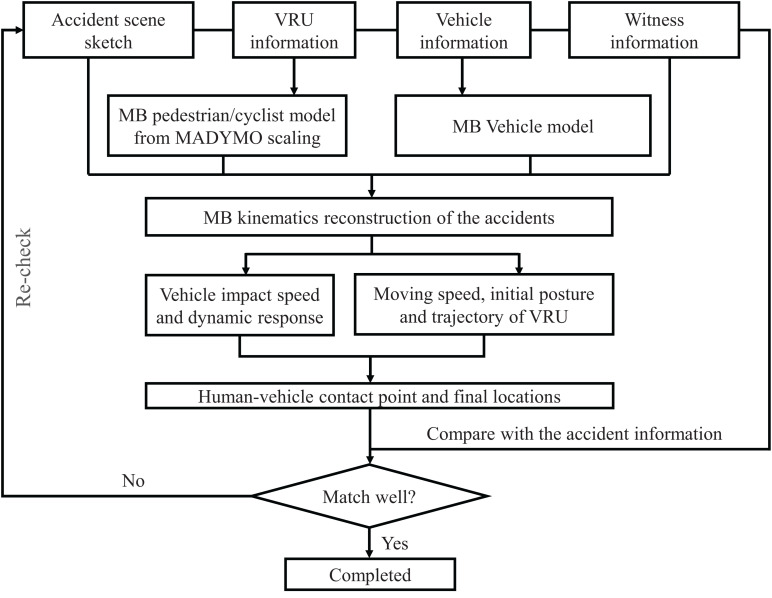
Flowchart of VRU traffic accident kinematics reconstruction. VRU, vulnerable road user.

The MB dynamics analysis software MADYMO is used to reconstruct the VRU impact accident. The MB human body model needed for accident reconstruction is obtained by scaling the TNO 50th percentile human body model introduced above according to human body information from the real accident using the Generator of Body Data (GEBOD) module in MADYMO software. The flowchart of VRU traffic accident reconstruction is shown in Figure 6.

**FIGURE 7:**
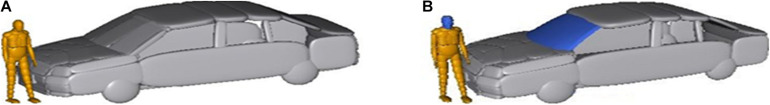
**(A)** MB model of vehicle and VRU and **(B)** coupled vehicle and VRU model. MB, multibody; VRU, vulnerable road user.

With the completion of the VRU traffic accident reconstruction, the MB human body model is replaced with the coupled FE–MB human body model described in section “Coupled Finite Element–Multibody Human Body Model,” and its posture is adjusted accordingly; meanwhile, the MB front windshield model is replaced with the FE model. According to the boundary conditions of car-to-VRU impact obtained from the MB kinematics reconstruction, the accident is re-simulated to obtain the head injury parameters. The MB model of the vehicle and the pedestrian in accident Case 11 is shown in Figure 7A, and the coupled FE–MB vehicle and pedestrian model is shown in Figure 7B as an example.

### Analysis of Effectiveness of Head Injury Evaluation Criteria

The head injury evaluation criteria and corresponding calculation methods employed in the present study, as shown in Table 3, were divided into two types: criteria based on head kinematics response (HIC, GAMBIT, BrIC, RIC, and HIP) and criteria based on brain tissue deformation (MPS, CSDM, and DDM). Among these criteria, HIC is most widely used in main stream vehicle safety standards/programs to evaluate the severity of head injury (Kleiven, 2007); however, this criterion solely relies on the linear kinematics of the head center of gravity (COG), without considering the influence of head rotational movement. The RIC is similar to the HIC, except that it uses rotational acceleration instead of linear acceleration. The GAMBIT and HIP criteria consider both the effects of linear and rotational acceleration of head COG. The BrIC considers the influence of maximum rotational velocity and maximum rotational acceleration. The CSDM measures the volume percentage of the area with brain strain exceeding a certain threshold in the whole brain volume, while DDM measures the volume percentage of the area with negative pressure exceeding a certain threshold in the whole brain.

**TABLE 3:** Evaluation criteria of head injury.

Evaluation criteria	Calculation method	Description
Head injury criterion, HIC (Versace, 1971b)	${\text{HIC}}_{15}={\left\{{\left[\left(t_{2}-t_{1}\right)\,\left(\frac{1}{\left(t_{2}-t_{1}\right)}\,\int_{t_{1}}^{t_{2}}a\,\left(t\right)\,\text{d}\,t\right)\right]}^{2.5}\right\}}_{\text{max}}$	a(t): Resultant linear acceleration of head centroid, g = 9.8 m/s^2^
Rotational injury criterion, RIC (Kimpara and Iwamoto, 2012)	$\text{RIC}={\left\{{\left[\left(t_{2}-t_{1}\right)\,\left(\frac{1}{\left(t_{2}-t_{1}\right)}\,\int_{t_{1}}^{t_{2}}\alpha \,\left(t\right)\,\text{d}\,t\right)\right]}^{2.5}\right\}}_{\text{max}}$	_α(t)_: Rotational acceleration of head centroid, rad/s^2^, *t*_2_-*t*_1_ = 36 ms
Generalized Acceleration Model for Brain Injury Threshold, GAMBIT (Newman, 1986)	$\text{GAMBIT}={\left[{\left(\frac{a_{\text{max}}}{a_{\text{cr}}}\right)}^{n}+{\left(\frac{\alpha_{\text{max}}}{\alpha_{\text{cr}}}\right)}^{m}\right]}^{\frac{1}{s}}$	_*a_max_*_ : Maximum linear acceleration, g; _*a_cr_*_ : Given the critical linear acceleration, its value is 350 ×g; _α_max__ : Maximum rotational acceleration, rad/s^2^; _α_cr__ : Given critical rotational acceleration, its value is 12,000 rad/s^2^
Head impact power, HIP (Marjoux et al., 2008)	$\text{HIP=}ma_{\text{x}}\;\int {\text{ a}}_{\text{X}}\text{d}t\text{ + }ma_{y}\;\int {\text{ a}}_{\text{y}}\text{d}t\text{ + }ma_{z}\;\int {\text{ a}}_{\text{z}}\text{d}t\;+\;I_{XX}\alpha_{X}\;\int \;\alpha_{X}\text{d}t\text{+}I_{yy}\alpha_{y}\;\int \;\alpha_{\text{y}}\text{d}t\text{ + }I_{zz}\alpha_{z}\;\int \;\alpha_{z}\text{d}t$	a_*x*_, a_*y*_, a_*z*_: translational acceleration, m/s^2^; α_*x*_, α_*y*_, α_*z*_: Rotational acceleration, rad/s^2^. In this study, the head centroid mass m = 4.5 kg; head centroid moment of inertia: *I*_*xx*_ = 0.016 kg/m^2^, *I*_*yy*_ = 0.024 kg/m^2^, *I*_*zz*_ = 0.022 kg/m^2^
Brain injury criterion, BrIC (Takhounts et al., 2013)	$\text{BrIC}=\frac{\omega_{\text{max}}}{\omega_{\text{cr}}}+\frac{\alpha_{\text{max}}}{\alpha_{\text{cr}}}$	_ω_max__ : Maximum rotational velocity, rad/s; _ω_cr__ : Given critical rotational velocity is 140 rad/s; _α_max__ : Maximum rotational acceleration rad/s^2^; _α_cr__ : Given critical rotational acceleration, its value is 12,000 rad/s^2^
Maximum principal strain, MPS (Gabler et al., 2016)	Used to predict diffuse axonal injury (DAI) and brain contusion	Measures the amount of strain in the tensile direction
Simulated Injury Monitor, SIMon (Takhounts et al., 2003)	Cumulative strain damage measure, CSDM; used to predict diffuse axon injury (DAI), with generally 15% or 25% as the threshold	Measures the volume percentage of the area with brain strain exceeding a certain threshold in the whole brain volume
Simulated Injury Monitor, SIMon (Takhounts et al., 2003)	Dilatation damage measure, DDM. To predict brain contusion and laceration, -100 kPa is generally set as the threshold of negative pressure	Measures the volume percentage of the area with negative pressure exceeding a certain threshold in the whole brain

Based on the selected 31 VRU traffic accident cases and subsequent reproduction of head injury as described above, the injury parameter values were calculated according to the formula of each criterion and compared with the Abbreviated Injury Scale/Maximum Abbreviated Injury Scale (AIS/MAIS) score for each accident head in Table 2.

Pearson’s correlation coefficient analysis method was used to analyze the effectiveness of each criterion in predicting head injury. Pearson’s correlation coefficient is a measure of the degree of linear correlation between variables, generally represented by the letter *r*. It is calculated by the product-difference method, which is based on the dispersion of two variables and their respective average values and reflects the correlation degree between two variables by multiplying the two dispersion values. The overall correlation coefficient of random variables *X* and *Y* is ρ(*X*,*Y*) = (Cov(*X*,*Y*))/(σ_*X*_.σ_*Y*_), where *Cov* (*X*, *Y*) is the covariance of *X* and *Y*,_  σ_X__ indicates the standard deviation of *X*, whereas_  σ_Y__ is the standard deviation of *Y*. However, the overall correlation coefficient ρ (*X*, *Y*) generally cannot be obtained, but only an estimate of ρ (*X*, *Y*) can be given according to the observed values of samples, which is called the sample correlation coefficient. Pearson’s correlation coefficient can be acquired by estimating the covariance and standard deviation of samples, which is often represented by *r*, and its expression is as follows:

(1) $$r=\frac{\sum_{i=1}^{n}\left(X_{i}-\bar{X}\right)\,\left(Y_{i}-\bar{Y}\right)}{\sqrt{\sum_{i=1}^{n}{\left(X_{i}-\bar{X}\right)}^{2}}\,\sqrt{\sum_{i=1}^{n}{\left(Y_{i}-\bar{Y}\right)}^{2}}}$$

In this formula, $\bar{X}$ and $\bar{Y}$ represent the average value of *X* and *Y*, respectively. The value of *r* is between -1 and 1. The greater the absolute value of *r*, the stronger the correlation between variable *x* and variable *y*. If *r* > 0, the correlation between the two variables is positive, whereas if *r* < 0, this correlation is negative.

Moreover, the current research particularly focuses on the investigation of brain injury prediction for the analyzed accidents by using HICs based on brain tissue deformation. As mentioned in section “Accident data,” two types of brain injury occurred in the selected 31 VRU accident cases: DAI and brain contusion (see Table 2). For DAI, CSDM (Takhounts et al., 2003, 2008, 2013; Gabler et al., 2016), and MPS (Takhounts et al., 2008; Gabler et al., 2016) injury criteria were analyzed; for brain contusion, DDM (Takhounts et al., 2003), and MPS (Bain and Meaney, 2000) injury criteria were investigated. Finally, the predicted injury criteria, in combination with the existing brain injury risk curves reported in the literature (Takhounts et al., 2003, 2008), were compared with the AIS scores of each accident in Table 2, and the effectiveness of each HIC in predicting human DAI and brain contusion in the accident was subsequently analyzed.

## Results

### Results of Vulnerable Road User Impact Accident Reconstruction

The results of the predicted kinematics response parameters of the accidents, including collision point between VRUs and vehicles, and the final rest positions, are consistent with the actual accident information. Using these kinematics reconstructions, we can obtain the initial boundary conditions of the accident cases, such as the impact velocities of both VRUs and vehicles, and the VRU trajectories.

**FIGURE 8:**
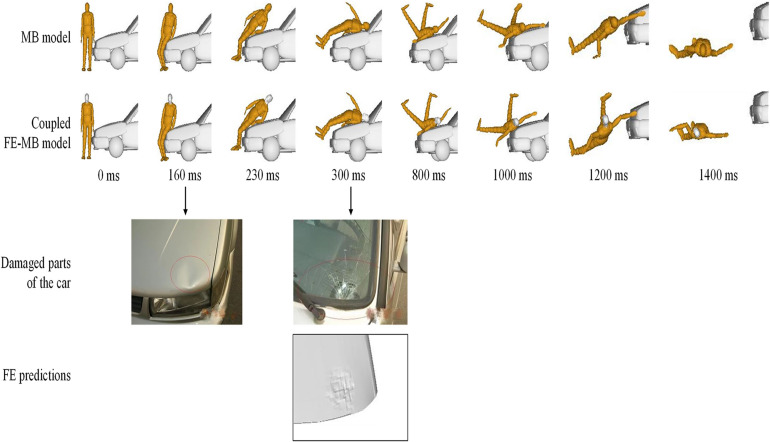
Predicted pedestrian kinematics and the comparison of vehicle deformation between accident reconstruction and real accident, with Case 1 as the example.

**FIGURE 9:**
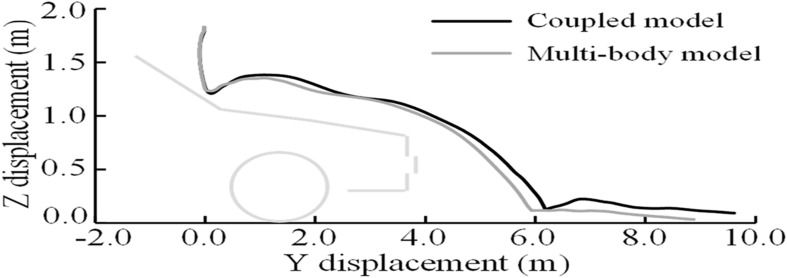
Comparison of the trajectory of VRU head COG in the YOZ plane in Case 1 between using MB and coupled FE–MB models. VRU, vulnerable road user; COG, center of gravity; MB, multibody; FE, finite element.

Taking Case 1 as an example, the kinematics responses of the pedestrian during the impact by using both MB and the coupled FE–MB models are shown in Figure 8. It can also be seen that the predicted damaged locations of the vehicle by FE method matches well with those in the real accident. Moreover, the trajectory of the head COG of the pedestrian in the YOZ plane (composed of vehicle moving Y-direction and vertical Z-direction) is also compared between the sole MB model and the coupled FE–MB model, as shown in Figure 9 and see Supplementary Appendix 1 for other cases.

### Analysis of Head Kinematics-Based Injury Criteria

On the basis of the description of the head kinematics-based injury criteria in section “Analysis of Effectiveness of Head Injury Evaluation Criteria” (HIC, GAMBIT, BrIC, RIC, and HIP), the injury criteria are calculated for each accident case (Table 4) and compared with the recorded MAIS scores in Table 2. Pearson’s correlation coefficient is used to analyze and evaluate the prediction and evaluation performance of each injury criterion for head injury.

**TABLE 4:** Calculated parametric values of head injury criteria.

Case ID	HIC	GAMBIT	BrIC	RIC	HIP (kW)
1	780.02	4.33	4.45	189,537,000	4.88
2	184.69	1.73	1.95	54,634,500	6.29
3	1,586.97	3.30	3.60	136,134,000	4.56
4	1,031.01	5.15	5.37	249,415,000	20.19
5	2,939.93	2.09	2.25	48,786,900	15.80
6	1,391.30	6.33	6.55	379,812,000	4.22
7	3,833.51	10.11	10.09	350,811,000	10.00
8	2,051.95	11.14	11.36	385,087,000	19.64
9	699.90	1.89	2.00	73,385,300	11.97
10	1,288.22	4.54	4.73	208,250,000	14.04
11	3,569.25	9.12	9.35	653,978,000	15.41
12	3,395.78	8.60	8.72	619,131,000	22.46
13	2,369.58	9.94	9.94	845,224,000	6.55
14	2,503.95	2.89	2.94	244,043,000	4.39
15	1,499.24	1.78	2.35	160,381,000	14.43
16	772.20	2.45	2.73	38,496,500	2.73
17	1,533.34	2.23	2.23	142,236,000	7.53
18	8,107.39	5.26	5.53	625,871,900	19.88
19	197.54	0.72	0.91	9,397,440	5.07
20	254.16	1.80	1.84	13,210,200	6.62
21	311.20	0.99	1.09	6,189,740	8.17
22	1,205.82	3.53	3.94	271,328,000	4.16
23	9,256.10	6.29	6.36	710,420,000	16.89
24	1,757.95	6.57	6.60	453,202,000	21.64
25	678.03	0.77	0.63	3,555,080	1.51
26	7,249.62	5.93	5.91	437,064,000	28.70
27	883.26	2.11	2.26	175,018,000	7.88
28	3,365.14	9.04	9.07	786,648,000	4.79
29	1,423.95	2.46	2.92	152,093,000	11.26
30	2,013.30	4.29	4.42	146,262,000	13.69
31	1,411.30	1.74	1.95	59,559,500	5.68

*HIC, head injury criterion; GAMBIT, Generalized Acceleration Model for Brain Injury Threshold; BrIC, brain rotational injury criterion; RIC, rotational injury criterion; HIP, head impact power.*

Assuming that *X* in formula (1) is the calculated value of HICs in each case, and *Y* in formula (1) is the MAIS of head injury, formula (1) is used to calculate the correlation coefficient of predicted criterion and head injury MAIS, as shown in Table 5. The results of correlation coefficient analysis seem to indicate that HIC, the most widely used injury criterion, has the best correlation with head injury MAIS, followed by HIP, RIC, BrIC, and GAMBIT, with the latter showing the worst correlation.

**TABLE 5:** Correlation coefficients between calculated injury criteria and MAIS.

Evaluation criterion	HIC	GAMBIT	BrIC	RIC	HIP
Correlation coefficient	0.606	0.332	0.340	0.398	0.403

*MAIS, Maximum Abbreviated Injury Scale; HIC, head injury criterion; GAMBIT, Generalized Acceleration Model for Brain Injury Threshold; BrIC, brain rotational injury criterion; RIC, rotational injury criterion; HIP, head impact power.*

**FIGURE 10:**
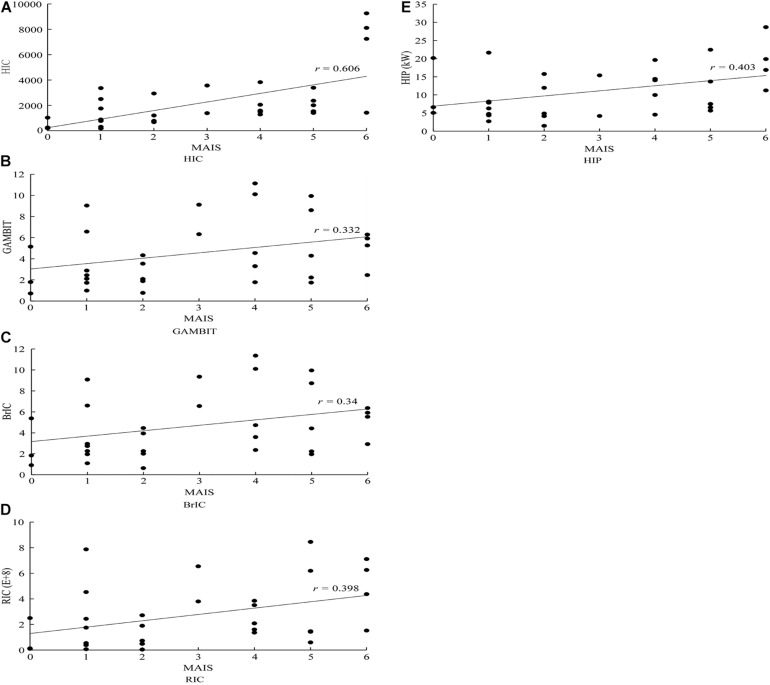
Distribution of predicted head kinematics-based injury criteria and MAIS scores observed in the analyzed VRU impact accident cases. **(A)** HIC; **(B)** GAMBIT; **(C)** BrIC; **(D)** RIC; and **(E)** HIP. MAIS, Maximum Abbreviated Injury Scale; VRU, vulnerable road user; HIC, head injury criterion; GAMBIT, Generalized Acceleration Model for Brain Injury Threshold; MAIS, Maximum Abbreviated Injury Scale; RIC, rotational injury criterion; HIP, head impact power.

The predicted HIC, GAMBIT, BrIC, RIC, HIP, and their corresponding MAIS in 31 VRU traffic accidents selected for this study are also graphically displayed in Figure 10.

### Analysis of Injury Criteria Based on Brain Tissue Deformation

Based on the actual DAI and brain contusion injury records observed in accidents, the HICs based on brain tissue deformation are also computed, which include CSDM (Takhounts et al., 2003, 2008, 2013; Gabler et al., 2016) (for DAI), MPS (Takhounts et al., 2008; Gabler et al., 2016) (for DAI and brain contusion), and DDM (Takhounts et al., 2003) (for brain contusion), as shown in Tables 6, 7.

**TABLE 6:** The AIS of DAI in accident cases included in this study and corresponding parametric values of head injury criteria.

Case ID	DAI AIS	CSDM_0.15_	CSDM_0.25_	MPS
1	2	0.98991727	0.850504137	0.881
2	1	0.82348759	0.389154602	0.603
3	3	0.840098242	0.362849018	0.556
4	0	0.998771975	0.942735264	1.29
5	2	0.997608583	0.916041882	1.019
6	3	0.998061013	0.990951396	2.457
7	4	0.999224405	0.944738883	2.32
15	4	0.993148914	0.985780765	1.402

*AIS, Abbreviated Injury Scale; DAI, diffuse axonal injury; MPS, maximum principal strain.*

**TABLE 7:** AIS score of brain contusion in the accident cases included in this study and the corresponding damage evaluation criteria parameter values.

Case ID	Brain contusion AIS	MPS	DDM
3	4	0.556	0
6	2	2.457	0.020488625
8	3	1.181	0.043110134
9	2	0.818	0.000129266
11	3	0.934	0.000129266
12	5	1.833	0.032445708
22	2	1.092	0.018420372

*AIS, Abbreviated Injury Scale; MPS, maximum principal strain; DDM, dilatation damage measure.*

**FIGURE 11:**
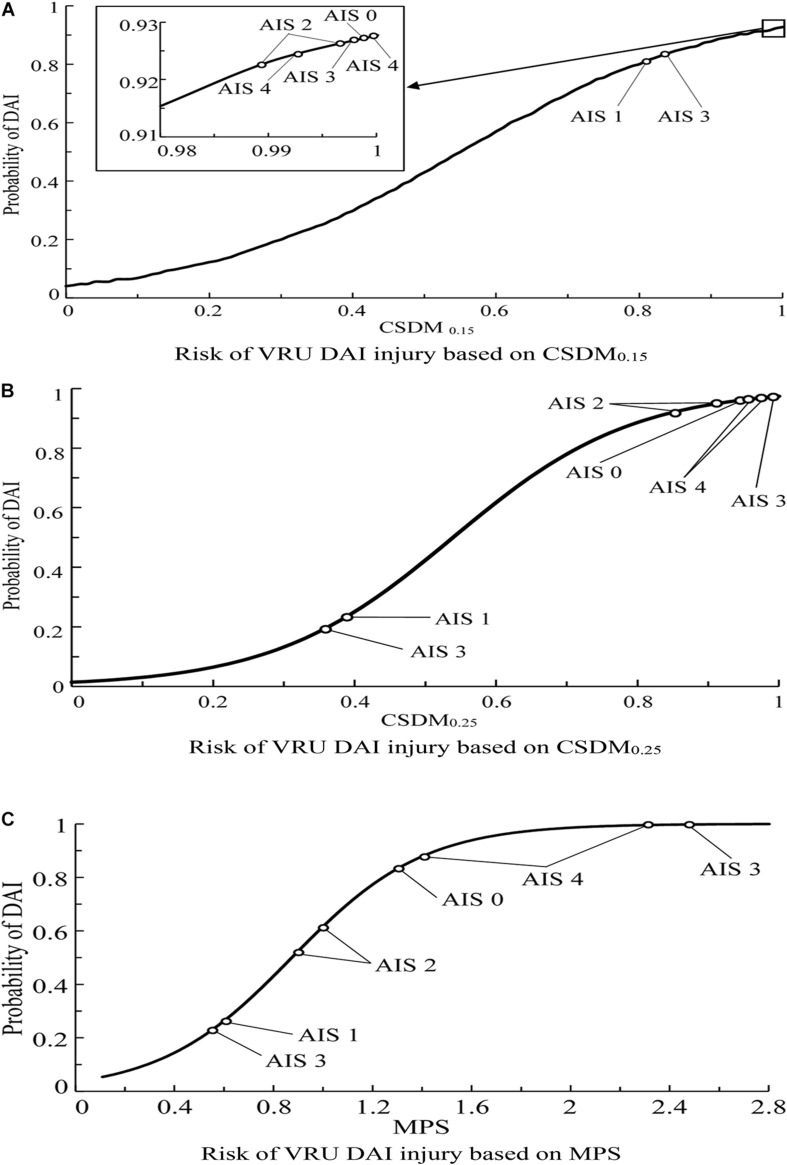
Predicted probability of the DAI occurrence for the VRU impact accident cases analyzed in this study using brain injury risk curves reported in the literature (Takhounts et al., 2003, 2008). **(A)** Risk of VRU DAI injury based on CSDM_0.15_, **(B)** Risk of VRU DAI injury based on CSDM_0.25_, and **(C)** Risk of VRU DAI injury based on MPS. DAI, diffuse axonal injury; VRU, vulnerable road user; MPS, maximum principal strain.

**FIGURE 12:**
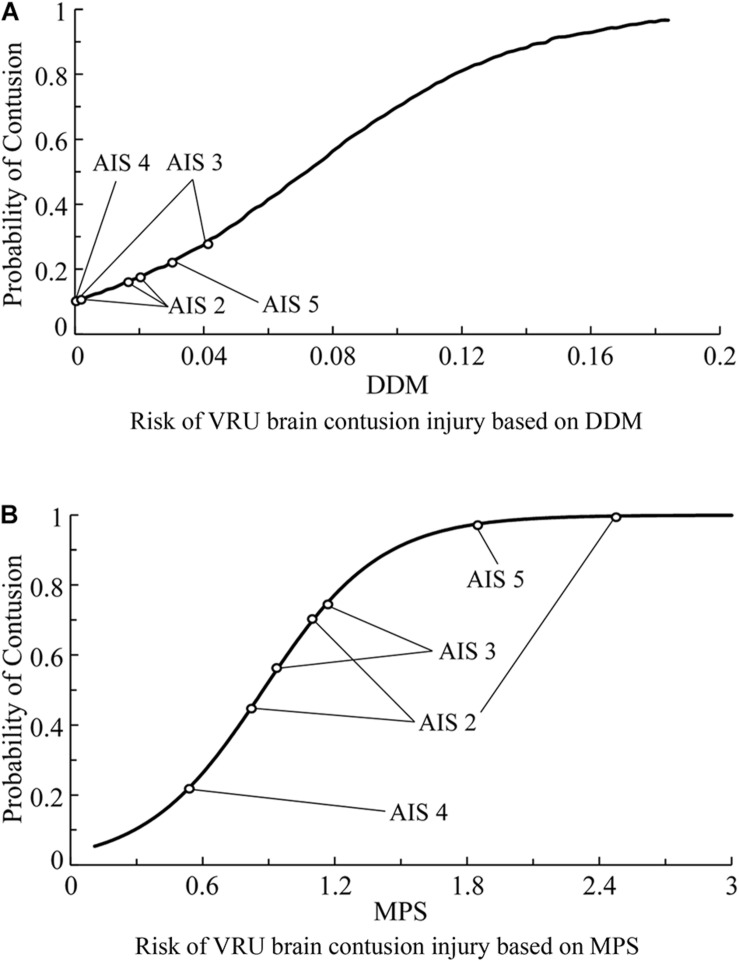
Predicted probability of the brain contusion occurrence for the VRU impact accident cases analyzed in this study using brain injury risk curves reported in the literature (Takhounts et al., 2003, 2008). **(A)** Risk of VRU brain contusion injury based on DDM and **(B)** Risk of VRU brain contusion injury based on MPS. VRU, vulnerable road user; DDM, dilatation damage measure.

In order to establish a relationship between the predicted injury criterion and the severity of actual brain injury, the brain injury risk curves in the existing literature are selected to analyze the effectiveness of the criteria in predicting brain injuries. Herein, the injury risk curve established by Takhounts et al. (2003, 2008) is selected for DAI. Combined with the predicted criterion in Table 6, the effectiveness of CSDM and MPS injury criteria in predicting DAI is analyzed separately, as shown in Figure 11. For brain contusion, the injury risk curve selected herein is that established by Takhounts et al. (2003, 2008). The calculated criterion values in Table 7 are used to analyze/evaluate the effectiveness of DDM and MPS in predicting brain contusion, as shown in Figure 12.

As seen in Figure 11A, the predicted brain injury risks based on CSDM_0.15_ for the eight accidents with DAI injury has no clear distribution pattern in relation to the DAI AIS scores; Figure 11B demonstrates that only the injury risks calculated from CSDM_0.25_ for the cases with AIS1 and AIS4 show certain regularity, those of the two cases with AIS3 scores are significantly different, and the injury risk corresponding to AIS0 is fairly high and close to that of AIS4 cases. In Figure 11C, the cases of AIS1, AIS2, and AIS4 show relatively appropriate uniformity along with the brain injury risk calculated from MPS. As for brain contusion, Figure 12A shows that the risk of brain contusion reflected by DDM injury criterion is far lower than the actual injury, implying that brain contusion cannot be predicted through DDM. For the brain contusion risk (Figure 12B), certain regularity can be observed in relation to the AIS scores for a case with AIS2 (the lower one) and cases with AIS3 and AIS5, while the risk corresponding to AIS4 case is relatively small.

From the analysis results above, it is suggested that the MPS injury criterion can better predict the possibility of DAI injury compared with CSDM_0.15_ and CSDM_0.25_, and it behaves better than DDM in predicting the risk of brain contusion.

## Discussion

### Coupled Finite Element–Multibody Human Body Model

In the present study, a coupled FE–MB human body model, developed and validated in our previous study, was used in the reconstruction of VRU impact accidents. The head trajectories predicted by using the MB model or the coupled FE–MB human body model (as shown in Figure 9 and Supplementary Appendix 1) appear to show a good match, especially before the VRU head contacts the windshield. This coupled model was initially proposed in our previous study to overcome the well-known limit of head-only FE model (representing only the pedestrian head and brain) in predicting pedestrian brain injury due to car impact and validated against a real-world car-to-pedestrian impact accident (Wang et al., 2020). That model was later further validated in our study (Yu et al., 2020) in which three cadaver experiments reported in the literature were reproduced with both the coupled body model and an MB body model, and the effectiveness of the coupled model was verified by comparing the pedestrian head kinematics and injury response produced by both models with the experimental results. In the current study, the coupled model was used to reconstruct all of the selected real-world VRU impact accidents and subsequently showed quite similar performance with the MB model in predicting the VRU kinematics, which also confirms its effectiveness.

The FE model has better biofidelity than the MB human body model in reflecting the biomechanical response of the human head in impact accidents (Chai et al., 2011). Nonetheless, the shortcomings of the FE human body model in the accident reconstruction are also clear: the calculation time is too long, the human body posture adjustment is overly complicated, and the time cost is relatively high. The coupled FE–MB human body model is introduced to address these issues, which can predict both the human body overall kinematics and brain soft tissue deformation responses and effectively improve the efficiency of collision simulation (by up to 82%) (Wang et al., 2020), thus avoiding frequent adjustments of the initial posture of the FE human body model during the reconstruction.

### Head Injury Criteria

In this study, 31 car-to-VRU impact accident cases with detailed head injury records were recruited to analyze/evaluate various HICs based on kinematics and brain tissue deformation. The evaluation of head kinematics-based injury criteria in predicting overall head injury was conducted by using Pearson’s correlation coefficient method (Tables 4, 5), and the brain deformation-based criteria were investigated using the brain injury risk curves in the existing literature combined with actual AIS scores (Figure 11).

The current study on the effectiveness of HICs in evaluating VRU head injury in traffic accidents is limited, and from the authors’ point of view, it is due to the fact that the number of analyzed samples is not big enough and that the lack of video information of part of the accident cases would potentially affect the kinematics reconstruction of the accident. Differences can be observed between the current study and those reported in the literature (Takhounts et al., 2008; Hernandez et al., 2015; Jones et al., 2016; Feng, 2017; Shi et al., 2020). Shi et al. (2020) conducted similar research based on real-world VRU (including pedestrians and cyclists) impact accident reconstruction using both MB and FE human body models, in which the human body impact boundary conditions at the time of ground landing were extracted from the MB kinematics reconstruction and input into a full-scale FE human body model for head injury reproduction. Their results indicated that HIC, MPS, and CSDM_0.15_ had the best ability to predict head injury, followed by CSDM_0.25_, HIP, BrIC, and DDM, with the latter having the worst ability. Jones et al. (2016) used the GHBMC 50th percentile adult male head–neck model to conduct a huge number of car-to-pedestrian impact simulations and obtain the head injury responses. Results on the analysis of the relationship between HIC and BrIC and brain injury severity suggested that the correlation between BrIC and brain contusion/DAI is higher than that of HIC. Takhounts et al. (2008) performed a similar research focusing on American football accidents and concluded that CSDM_0.25_ and MPS had good correlations with DAI, whereas DDM was not related to contusion or focal lesion. Hernandez et al. (2015) demonstrated that MPS had the best ability to predict mild TBI (MTBI), followed by HIP and GAMBIT. Feng (2017) used head-only FE model to predict pedestrian head/brain injury responses during the head-windscreen impact of a real-world accident and found that MPS had the best ability to predict DAI.

In the present work, HIC and HIP injury criteria were shown to have the best correlation with MAIS, which is consistent with the study by Shi et al. (2020). In comparison with CSDM_0.15_ and CSDM_0.25_, the MPS damage criterion appeared to better predict the occurrence of DAI injury, which is in good agreement with the conclusions of Takhounts et al. (2008) and Feng (2017). With regard to predicting brain contusion, MPS can provide better ability than DDM, and this finding is consistent with the research by Takhounts et al. (2008). Meanwhile, differences exist between the current research and those reported in the literature, especially for the evaluation of brain deformation-based injury criteria. This can be explained by the application of the coupled FE–MB human body model that accounted for the influences of the rest of the human body on the head kinematics/injury responses compared with FE head-only models (Wang et al., 2020), which we believe could bring more confidence about the novelty of this study in the accuracy of HICs.

### Limitations

The main limitation of this study is that the number of reconstructed traffic accident cases is limited, and the methods of accident reconstruction are backward, which may lead to potential variations in the behavior of certain injury criteria. Moreover, since many factors influence the kinematics reconstruction of the accident, such as VRU initial posture, thereby it cannot be guaranteed that the simulation accurately reproduces the actual accidents, leading to a potential impact on the accuracy of head injury analysis. Lastly, the head–neck FE model used in this study is extracted from the THUMS model, which represents the 50th percentile adult male. In fact, the biomechanical properties of the head tissues of adults and the elderly are different, which leads to different injury tolerance and kinematics response by different ages.

## Conclusion

In this work, the coupled FE–MB human body model was used to simulate VRU injury in real traffic accidents, kinematics reconstruction, and head/brain injury reproduction of a series of real-world car-to-VRU impact accidents to investigate the effectiveness of various HICs in predicting the head injury risk due to VRU–car collision. According to the results, the following conclusions can be drawn:

1. The coupled FE–MB human body model can efficiently and accurately simulate the kinematics response and head/brain injuries of VRUs in impact accidents and can be effectively used for the analysis of head/brain injury due to VRU–car collision.
2. Among the injury criteria based on head kinematics response, the most widely used HIC and HIP are the most accurate and effective criteria in predicting head injury. Considering brain tissue deformation-based injury criteria, the MPS injury criterion can more effectively predict the possibility of DAI than the CSDM_0.15_ and CSDM_0.25_. For brain contusion, the MPS injury criterion shows enhanced ability to predict the injury risk compared with the DDM criterion.

## Data Availability Statement

The original contributions presented in the study are included in the article/Supplementary Material, further inquiries can be directed to the corresponding author.

## Author Contributions

FW: conceptualization, project administration, methodology, funding acquisition, and writing—review and editing. ZW: software and writing—original draft. LH: supervision and funding acquisition. HX: formal analysis and visualization. CY: validation and methodology. FL: investigation and resources. All authors contributed to the article and approved the submitted version.

## Conflict of Interest

The authors declare that the research was conducted in the absence of any commercial or financial relationships that could be construed as a potential conflict of interest.

## Footnotes

1 http://www.lstc.com

2 https://www.tassinternational.com/madymo

3 https://www.tno.nl/en/

4 https://www.toyota.co.jp/thums/

## Supplementary Material

Supplementary Appendix 1 Comparison of the trajectory of VRU head COG in the YOZ plane in Cases 2–31 when using MB or coupled FE–MB models. VRU, vulnerable road user; COG, center of gravity; MB, multibody; FE, finite element.

## References

[B1] AAAM (2008). *Abbreviated Injury Scale 2005,Update 2008.* Barrington: Association for Advancement of Automatic Medicine.

[B2] Al-Bsharat A. S. Hardy W. N. Yang K. H. Khalil T. B. Tashman S. King A. I. (1999). *Brain/Skull Relative Displacement Magnitude Due to Blunt Head Impact: New Experimental Data and Model (No. 99SC22).* Warrendale: SAE.

[B3] Antona-Makoshi J. (2016). *Traumatic Brain Injuries: Animal Experiments and Numerical Simulations to Support the Development of a Brain Injury Criterion.* Sweden: Chalmers University of Technology Gothenburg.

[B4] Bain A. C. Meaney D. F. (2000). Tissue-level thresholds for axonal damage in an experimental model of central nervous system white matter injury. *J. Biomech. Eng.* 122 615–622. 10.1115/1.1324667 11192383

[B5] Chai X. Jin X. Zhang X. Hou X. (2011). The application for skull injury in vehicle–pedestrian accident. *Int. J. Crashworthiness* 16 11–24.

[B6] Corrigan J. D. Selassie A. W. Orman J. A. L. (2010). The epidemiology of traumatic brain injury. *J. Head Trauma Rehabil.* 25 72–80. 20234226 10.1097/HTR.0b013e3181ccc8b4

[B7] Deguchi M. (2003). “Modeling of a motorcycle for collision simulation,” in *Proceedingsof the International Technical Conference on the Enhanced Safety of Vehicles*, Gothenburg.

[B8] Fahlstedt M. Halldin P. Alvarez V. Kleiven S. (2016). “Influence of the body and neck on head kinematics and brain injury risk in bicycle accident situations. *Paper Presented at the IRCOBI 2016*, (Zurich: IRCOBI).

[B9] Feng C. (2017). *Prediction of Pedestrian Death Risk and Brain Injury Type in Vehicle Collision.* Chongqing: Third Military Medical University.

[B10] Gabler L. F. Crandall J. R. Panzer M. B. (2016). Investigating brain injury tolerance in the sagittal plane using a finite element model of the human head. *Int. J. Automotive Eng.* 7 37–43. 10.1089/neu.2016.4758 29848152 PMC6444911

[B11] Gadd C. W. (1966). *Use of a Weighted-Impulse Criterion for Estimating Injury Hazard (No 660793)*. Warrendale: SAE.

[B12] Hernandez F. Wu L. C. Yip M. C. Laksari K. Hoffman A. R. Lopez J. R. (2015). Six degree-of-freedom measurements of human mild traumatic brain injury. *Ann. Biomed. Eng.* 43 1918–1934. 25533767 10.1007/s10439-014-1212-4 PMC4478276

[B13] Hu J. Jin X. Lee J. B. Zhang L. Chaudhary V. Guthikonda M. (2007). Intraoperative brain shift prediction using a 3D inhomogeneous patient-specific finite element model. *J. Neurosurg.* 106 164–169. 10.3171/jns.2007.106.1.164 17236503

[B14] Hu L. Bao X. Lin M. Yu C. Wang F. (2021). Research on risky driving behavior evaluation model based on CIDAS real data. *Proc. Instit. Mech. Eng. Part D J. Automobile Eng.* 235:095440702098597. 10.1177/0954407020985972

[B15] Hu L. Hu X. Wang J. Kuang A. Hao W. Lin M. (2020). Casualty risk of e-bike rider struck by passenger vehicle using China in-depth accident data. *Traf. Injury Prevent.* 21 283–287. 10.1080/15389588.2020.1747614 32297809

[B16] Jones D. A. Urban J. E. Weaver A. A. Stitzel J. D. (2016). “Investigation of head injury mechanisms through multivariate finite element simulation,” in *Proceedings of the 12th Ohio State University Injury Biomechanics Symposium*, Columbus, OH.

[B17] Katsuhara T. Miyazaki H. Kitagawa Y. Yasuki T. (2014). “Impact kinematics of cyclist and head injury mechanism in car-to-bicycle collision,” in *Proceedings of the IRCOBI conference 2014*, Zurich.

[B18] Kimpara H. Iwamoto M. (2012). Mild traumatic brain injury predictors based on angular accelerations during impacts. *Ann. Biomed. Eng.* 40 114–126. 21994065 10.1007/s10439-011-0414-2

[B19] Kleiven S. (2007). *Predictors for Traumatic Brain Injuries Evaluated Through Accident Reconstructions (No. 2007-22-0003).* Warrendale: SAE. 10.4271/2007-22-0003 18278592

[B20] Kong C. Yang J. (2010). Logistic regression analysis of pedestrian casualty risk in passenger vehicle collisions in China. *Accident Anal. Prevent.* 42 987–993. 10.1016/j.aap.2009.11.006 20441804

[B21] Li F. Liu N. S. Li H. G. Zhang B. Tian S. W. Tan M. G. (2019). A review of neck injury and protection in vehicle accidents. *Transp. Saf. Environ.* 1 89–105.

[B22] Li F. Yang J. (2010). A study of head–brain injuries in car-to-pedestrian crashes with reconstructions using in-depth accident data in China. *Int. J. Crashworthiness* 15 117–124.

[B23] Li G. Yang J. Simms C. (2017). Safer passenger car front shapes for pedestrians: a computational approach to reduce overall pedestrian injury risk in realistic impact scenarios. *Accident Anal. Prevent.* 100 97–110. 10.1016/j.aap.2017.01.006 28129577

[B24] Lissner H. Lebow M. Evans F. (1960). Experimental studies on the relation between acceleration and intracranial pressure changes in man. *Surg. Gynecol. Obstetr.* 111:329. 14417481

[B25] Lyons M. Simms C. K. (2012). “Predicting the influence of windscreen design on pedestrian head injuries,” in *Paper presented at the IRCOBI Conference*, (Zurich: IRCOBI).

[B26] Marjoux D. Baumgartner D. Deck C. Willinger R. (2008). Head injury prediction capability of the HIC, HIP, SIMon and ULP criteria. *Accident Anal. Prevent.* 40 1135–1148. 10.1016/j.aap.2007.12.006 18460382

[B27] Miller R. T. Margulies S. S. Leoni M. Nonaka M. Chen X. Smith D. H. (1998). Finite element modeling approaches for predicting injury in an experimental model of severe diffuse axonal injury. *SAE Trans.* 4 2798–2810.

[B28] Nahum A. M. Smith R. Ward C. C. (1977). *Intracranial Pressure Dynamics During Head Impact (No. 770922).* Warrendale: SAE.

[B29] Newman J. Barr C. Beusenberg M. C. Fournier E. Shewchenko N. Welbourne E. (2000). “A new biomechanical assessment of mild traumatic brain injury. Part 2: results and conclusions,” in *Proceedings of the International Research Council on the Biomechanics of Injury conference*, Zurich. 10.1227/01.neu.0000196265.35238.7c

[B30] Newman J. A. (1986). “A generalized acceleration model for brain injury threshold (GAMBIT),” in *Proceedings of the 1986 International Research Council on the Biomechanics of Injury Conference*, Zurich. 10.1007/s10439-019-02382-2

[B31] Nie J. Yang J. (2014). A study of bicyclist kinematics and injuries based on reconstruction of passenger car–bicycle accident in China. *Accident Anal. Prevent.* 71 50–59. 10.1016/j.aap.2014.04.021 24880929

[B32] Nie J. Yang J. (2015). A study on the dynamic response and injury of cyclist based on car-bicycle accident reconstruction. *Automotive Eng.* 37 160–166.

[B33] Nie J. Li G. Yang J. (2015). A study of fatality risk and head dynamic response of cyclist and pedestrian based on passenger car accident data analysis and simulations. *Traffic Inj. Prevent.* 16 76–83. 10.1080/15389588.2014.881477 24571385

[B34] Popescu C. Anghelescu A. Daia C. Onose G. (2015). Actual data on epidemiological evolution and prevention endeavours regarding traumatic brain injury. *J. Med. Life* 8:272. PMC4556905 26351526

[B35] Ruan S. Li H. Wang X. Liu W. (2007). A new exploration of the applicability and usability of criteria for judging head injury. *J. Biomed. Eng.* 24 1373–1377. 18232496

[B36] Shi L. Han Y. Huang H. Davidsson J. Thomson R. (2020). Evaluation of injury thresholds for predicting severe head injuries in vulnerable road users resulting from ground impact via detailed accident reconstructions. *Biomech. Model. Mechanobiol.* 19 1845–1863. 10.1007/s10237-020-01312-9 32133546

[B37] Shi L. Han Y. Huang H. Li Q. Wang B. Mizuno K. (2018). Analysis of pedestrian-to-ground impact injury risk in vehicle-to-pedestrian collisions based on rotation angles. *J. Saf. Res.* 64 37–47. 10.1016/j.jsr.2017.12.004 29636168

[B38] Shigeta K. Kitagawa Y. Yasuki T. (2009). “Development of next generation human FE model capable of organ injury prediction,” in *Proceedings of the 21st Annual Enhanced Safety of Vehicles*, Zurich.

[B39] Takhounts E. G. Craig M. J. Moorhouse K. McFadden J. Hasija V. (2013). Development of brain injury criteria (BrIC). *Stapp. Car Crash J.* 57 243–266. 24435734 10.4271/2013-22-0010

[B40] Takhounts E. G. Eppinger R. H. Campbell J. Q. Tannous R. E. Power E. D. Shook L. S. (2003). On the development of the SIMon finite element head model. *Stapp. Car Crash J.* 47 107–133. 17096247 10.4271/2003-22-0007

[B41] Takhounts E. G. Ridella S. A. Hasija V. Tannous R. E. Campbell J. Q. Malone D. (2008). *Investigation of Traumatic Brain Injuries using the Next Generation of Simulated Injury Monitor (SIMon) Finite Element Head Model (No. 2008-22-0001).* Warrendale: SAE. 10.4271/2008-22-0001 19085156

[B42] TASS (2013a). *Coupiling Manual. version 7.5.* Netherlands: TASS.

[B43] TASS (2013b). *MADYMO Human Body Models Manual Release 7.5.* Netherlands: TASS.

[B44] TASS (2013c). *MADYMO Manual Version 7.5.* Netherlands: TASS.

[B45] Versace J. (1971a). *A Review of the Severity Index (0148–7191).* Warrendale: SAE.

[B46] Versace J. (1971b). “A review of the severity index,” in *Proceedings of the 15th Stapp Car Crash Conference*, Coronado.

[B47] Wang F. Han Y. Wang B. Peng Q. Huang X. Miller K. (2018). Prediction of brain deformations and risk of traumatic brain injury due to closed-head impact: quantitative analysis of the effects of boundary conditions and brain tissue constitutive model. *Biomech. Model. Mechanobiol.* 17 1165–1185. 10.1007/s10237-018-1021-z 29754317

[B48] Wang F. Yu C. Wang B. Li G. Miller K. Wittek A. (2020). Prediction of pedestrian brain injury due to vehicle impact using computational biomechanics models: are head-only models sufficient? *Traffic Inj. Prevent.* 21 102–107. 10.1080/15389588.2019.1680837 31770038

[B49] Watanabe R. Miyazaki H. Kitagawa Y. Yasuki T. (2011). “Research of collision speed dependency of pedestrian head and chest injuries using human FE model (THUMS version 4),” in *Proceedings of the 22nd Int. Technical Conf. on the Enhanced Safety of Vehicles (ESV)*, Zurich.

[B50] Wittek A. Grosland N. M. Joldes G. R. Magnotta V. Miller K. (2016). From finite element meshes to clouds of points: a review of methods for generation of computational biomechanics models for patient-specific applications. *Ann. Biomed. Eng.* 44 3–15. 10.1007/s10439-015-1469-2 26424475

[B51] Yang J. (2005). Review of injury biomechanics in car-pedestrian collisions. *Int. J. Vehicle Saf.* 1 100–117.

[B52] Yang K. H. Mao H. Wagner C. Zhu F. Chou C. C. King A. I. (2011). “Modeling of the brain for injury simulation and prevention,” in *Biomechanics of the Brain*, ed. Miller K. (New York, NY: Springer), 99–110.

[B53] Yang Y. Chang T. Luo T. Li L. Qu Y. (2017). Research progress of multimodal monitoring in the treatment of severe traumatic brain injury. medical review. *Med. Life* 23 1346–1349.

[B54] Yao J. Yang J. Otte D. (2008). Investigation of head injuries by reconstructions of real-world vehicle-versus-adult-pedestrian accidents. *Saf. Sci.* 46 1103–1114.

[B55] Yu C. Wang F. Wang B. Li G. Li F. (2020). A computational biomechanics human body model coupling finite element and multibody segments for assessment of head/brain injuries in car-to-pedestrian collisions. *Int. J. Environ. Res. Public Health* 17:492. 10.3390/ijerph17020492 31941003 PMC7014246

