# Supplementary material for: Evaluation of Head Injury Criteria for Injury Prediction Effectiveness: Computational Reconstruction of Real-World Vulnerable Road User Impact Accidents
Source: Front Bioeng Biotechnol. 2021 Jun 29;9:677982. doi: 10.3389/fbioe.2021.677982 (PMC8275938; doi:10.3389/fbioe.2021.677982)
Supplement: Supplementary Appendix 1 — Comparison of the trajectory of VRU head COG in the YOZ plane in Cases 2–31 when using MB or coupled FE–MB models. VRU, vulnerable road user; COG, center of gravity; MB, multibody; FE, finite element. [file Data_Sheet_1.doc]

**Appendix**

Case 1 Case 2

Case 3 Case 4

Case 5 Case 6

Case 7 Case 8

Case 9 Case 10

Case 11 Case 12

Case 13 Case 14

Case 15 Case 16

Case 17 Case 18

Case 19 Case 20

Case 21 Case 22

Case 23 Case 24

Case 25 Case 26

Case 27 Case 28

Case 29 Case 30

Case 31

Appendix 1. Comparison of the trajectory of VRU head COG in the YOZ plane in Cases 2-31 when using MB or coupled FE-MB models.
